# Tropical pitcher plants (*Nepenthes*) act as ecological filters by altering properties of their fluid microenvironments

**DOI:** 10.1038/s41598-020-61193-x

**Published:** 2020-03-10

**Authors:** Kadeem J. Gilbert, Leonora S. Bittleston, Wenfei Tong, Naomi E. Pierce

**Affiliations:** 1 Department of Organismic and Evolutionary Biology, Harvard University, 26 Oxford St., Cambridge, MA 02138 USA; 2 Department of Civil and Environmental Engineering, Massachusetts Institute of Technology, 77 Massachusetts Avenue, Room 1-290, Cambridge, MA 02139 USA; 3 Department of Biological Sciences, Boise State University, 1910 W. University Drive, Boise, ID 83725 USA; 4 Department of Biological Sciences, University of Alaska, Anchorage, 3211 Providence Drive, Anchorage, AK 99508 USA

**Keywords:** Ecology, Microbiology

## Abstract

Characteristics of host species can alter how other, interacting species assemble into communities by acting as ecological filters. Pitchers of tropical pitcher plants (*Nepenthes*) host diverse communities of aquatic arthropods and microbes in nature. This plant genus exhibits considerable interspecific diversity in morphology and physiology; for example, different species can actively control the pH of their pitcher fluids and some species produce viscoelastic fluids. Our study investigated the extent to which *Nepenthes* species differentially regulate pitcher fluid traits under common garden conditions, and the effects that these trait differences had on their associated communities. Sixteen species of *Nepenthes* were reared together in the controlled environment of a glasshouse using commonly-sourced pH 6.5 water. We analyzed their bacterial and eukaryotic communities using metabarcoding techniques, and found that different plant species differentially altered fluid pH, viscosity, and color, and these had strong effects on the community structure of their microbiota. *Nepenthes* species can therefore act as ecological filters, cultivating distinctive microbial communities despite similar external conditions, and blurring the conceptual line between biotic and abiotic filters.

## Introduction

Living organisms can act as ecological filters that alter the ability of other species to establish and persist in defined local environments. The term “ecological filter” has predominantly been used in plant community ecology^1^; however, it can also be applied to any system in which some key ecological factor influences the assembly of the local community, regardless of which taxon comprises that community (e.g. plants^2^, insects^3^, vertebrates^4^, and microbes^5^). Also, the term’s use is not always strictly limited to biotic filtering agents (e.g. woody debris^6^, vertebrate carcasses^7^, and landscape heterogeneity^8^). Here, we apply the term ecological filter to the phytotelmata (plant-held waters) of tropical pitcher plants (*Nepenthes* L.: Nepenthaceae: Caryophyllales). These carnivorous plants have modified leaves known as ‘pitchers’ that contain a pool of plant secretions and rainwater used for the capture and digestion of arthropod prey^9^. Additionally, however, this digestive fluid serves as a habitat for a diverse community of symbiotic dipteran larvae, mites, bacteria, fungi, and protists^10–13^.

Many studies treat biotic and abiotic filters as conceptually separate, distinguishing between abiotic (often termed “environmental”) and biotic filters^14,15^. However, such a distinction may not always be a useful simplification. Abiotic factors, such as pH or oxygen availability, do not only affect organisms; the reverse can also occur through “ecological niche construction”^16,17^. For example, during the Great Oxygenation Event cyanobacterial photosynthesis led to a large increase in oxygen levels in Earth’s atmosphere. The distinction between biotic and abiotic influence also becomes blurred in small, contained environments such as inside termite mounds, where the inhabitants’ metabolic products strongly impact the entire system^18,19^. Biotic-abiotic interactions can have an even larger impact when the contained environment is within a living organism; in this case, the host’s “extended phenotype”^20^ can directly influence the entire inhabitant community and define the bulk of the abiotic conditions experienced, especially if the host has evolved to regulate those conditions in specific ways.

Because of this, the ecological filter concept is of considerable utility to the study of host-microbiome interactions. A general concept of ecological filters that recognizes the integrated nature of abiotic and biotic factors can be useful in inquiring into the nature of microbial community assembly: to what extent can closely related host species produce characteristically distinguishable microbiota given identical external environmental conditions?

The genus *Nepenthes* contains over 140 described species and the pitchers of different species exhibit a high level of morphological diversity, varying widely in shape, size, and coloration^21,22^, which can correspond to diverse dietary specializations^23–27^. Physiological traits related to the abiotic conditions of pitcher fluid also can vary among species^28^. Interspecific trait differences in pitchers may mediate their interactions with symbionts and affect the resultant community composition of the microbiome. Multiple studies have shown that different plant species^29,30^ or even genotypes within species^31^ can have distinguishable microbiota when placed in a common environment. Due to the nature of the digestive fluid, however, pitcher pools could potentially exert stronger selective control over the microbiota contained within.

One possible means of selective control is via the biochemistry of the fluid. Pitchers secrete a variety of digestive enzymes including proteases, chitinases, glucanases, glucosidases, nucleases, esterases, peroxidases, phosphatases, and ureases^32,33^. The aspartic proteases nepenthesin I and nepenthesin II are characteristic of *Nepenthes*^34,35^. Additionally, pitchers secrete compounds with anti-bacterial^36^ and anti-fungal properties^37^ that could directly constrain which taxa can colonize and establish in these environments. A number of other fluid traits may also act as ecological filters. For instance, pitchers alter the viscosity of their fluid: some species produce highly viscoelastic fluid, well adapted to increase prey retention^38–41^. Viscosity can differentially affect bacterial taxa, as has been seen in animal gut environments^42–44^ and motor oil^45^; the effect of viscosity on flagellar motility^46^ may well favor the growth of certain taxa and thus may have broad effects on a community level.

Pitchers can also actively regulate their pH levels, with likely species differences in this trait^47^. Individual species of bacteria are typically constrained to specific pH ranges^48^ and given the strong effect of pH in structuring microbial communities in a wide variety of other contexts, including roots^49–51^, soils^52^, and guts^53^, it is highly likely pH can act as a filter for pitchers. Indeed, prior studies of *Nepenthes* microbiota indicate that pH can be a major factor in shaping communities^54,55^ and field studies also point to interspecific differences in fluid pH^56,57^.

The goal of the present study is to determine the extent to which *Nepenthes* species differentially regulate pitcher fluid traits given common rearing conditions, and the effect that such interspecific trait differences have on community assembly of the microbiota. This study takes advantage of the highly controlled environmental conditions within a horticultural glasshouse. We used plants reared in a *Nepenthes* nursery at Singapore’s “HortPark”. This nursery contained a large collection of *Nepenthes* species with geographic origins across the distribution of the genus; plants were propagated in a common garden setting within a climate-controlled glasshouse.

An earlier study^54^ similarly utilized a common garden setup to explore ecological filtering in 7 local *Nepenthes* species found in Thailand. Our study is an advance over previous work in that more species (15) were studied in a more controlled experimental setting: plants kept fully indoors, reducing exposure to a large uncharacterized potential arthropod pool and variable external conditions. We filled pitchers with water from a common source and standardized pH in order to maximize the similarity of the starting conditions, thereby increasing confidence that any resulting differences in fluid properties and microbiome communities could be attributed to plant physiological traits. Although artificial in nature, this study provides novel data on the *Nepenthes* microbiome that can be compared with results from previous analyses and improve our understanding of wild community assembly, as well as the potential power of trait-based ecological filtering.

We emphasize that it was not our intention to investigate the ecology of this particular set of *Nepenthes* species and the composition of their symbiotic communities in their natural habitats. Rather, we were interested in determining the extent to which different host species are able to differentially filter a common microbial pool under common rearing conditions. Thus, we designed the experiment to reduce external environmental variation as much as possible. Another objective was to maximize the likelihood of observing possible species differences by selecting as wide an assortment of species as possible; meeting this goal led to limitations in within-species replication. We focused on broad variance in traits across samples and a large number of samples overall, and to this end, we analyzed 86 pitcher fluid samples from 15 species and one hybrid. We expected to see differences in the fluid pH and viscosity among *Nepenthes* species housed in a common environment, and hypothesized that these differences would alter associated microbial communities.

## Materials and Methods

### Rearing conditions

The present study used plants cultivated for horticultural purposes in a dedicated *Nepenthes* glasshouse at the HortPark nursery in Singapore. The 15 species (and one hybrid) of *Nepenthes* were originally imported to HortPark in 2014 as micropropagated clones from Borneo Exotics (Pvt) Ltd., a nursery located in Sri Lanka. The plants were grown together in the HortPark glasshouse under common conditions and thus were all roughly the same age at the time that their pitchers were sampled. The plants were potted in *Sphagnum* medium and foliar-fed using Gaviota 63 fertilizer. The glasshouse environment was regulated to mimic conditions representative of the natural habitat of high-altitude *Nepenthes* species: the temperature was kept at 16 °C and the humidity at 80% relative humidity, maintained via an automatic misting system. The sealed indoor environment largely precluded entry by arthropods; however, fungus gnats (Sciaridae) could be found living in the *Sphagnum* medium. The water source used for misting and watering the plants was tap water that was filtered and mixed with enough hydrochloric acid to reach a target pH of 6.5. This pH-altered water was added to the pitchers *ad libitum* (“topping off” any endogenous fluid produced by the plant and counteracting evaporation and spills) such that they remained one-third full of fluid, which is a fluid level typically seen in wild pitchers. Maintaining relatively similar fluid levels in this way thus effectively reduced variation due to fluid volume (Table 1); we confirmed that volume lacked a significant effect on community composition for either bacteria or eukaryotes (Mantel tests, bacteria: r = 0.006, p = 0.44; eukaryotes: r = −0.009, p = 0.55). Water addition was ceased for two weeks prior to sampling in order to allow time for pitchers to adjust fluid properties.

**Table 1:** Summary of Nepenthes species sampled for this study.

Name	Region of Origin	Pitcher Morph	Total no. of plants	Mean no. pitchers sampled per plant ± standard deviation (sd)	Mean ± sd pH	Mean ± sd Fluid Volume (mL)	Mean ± sd Pitcher Length (mm)
*Nepenthes boschiana*	Borneo	L/I	2	3 ± 0	3.9 ± 1.2	5.93 ± 2.51	136.83 ± 9.67
*Nepenthes copelandii*	Philippines	L	2	2 ± 0	3.8 ± 1.5	7.6 ± 1.02	153.41 ± 33.08
*Nepenthes dubia*	Sumatra	U	3	1.67 ± 0.58	1.8 ± 0.8	0.48 ± 0.39	40.94 ± 3.94
*Nepenthes eymae*	Sulawesi	U + L^a^	2	1.50 ± 0.71	3.7 ± 1.2	1.9 ± 0.26	78.93 ± 10.46
*Nepenthes fusca*	Borneo	I	3	2.33 ± 0.58	4.9 ± 0.4	1.98 ± 0.30	139.66 ± 12.79
*Nepenthes hamata*	Sulawesi	L	2	1 ± 0	3 ± 1.4	1.76 ± 2.45	86.66 ± 30.56
*Nepenthes inermis*	Sumatra	U	3	2 ± 0	2.2 ± 0.4	0.66 ± 0.27	58.48 ± 27.18
*Nepenthes jacquelineae*	Sumatra	L	4	1.25 ± 0.50	2 ± 1.0	1.57 ± 0.40	45.08 ± 25.86
*Nepenthes khasiana*	India	U	2	1.50 ± 0.71	3.3 ± 0.6	1.33 ± 1.04	102.93 ± 10.98
*Nepenthes maxima*	Sulawesi & New Guinea	L^b^	4	1.75 ± 0.50	3 ± 0.8	4.29 ± 1.32	100.29 ± 32.99
*Nepenthes ramispina*	Malayan Peninsula	U	2	2 ± 0	2.3 ± 0.5	1.4 ± 0.47	109.71 ± 18.03
*Nepenthes sanguinea*	Malayan Peninsula	I	3	1.67 ± 0.58	2 ± 0.7	1.38 ± 0.16	107.44 ± 33.98
*Nepenthes singalana*	Sumatra	L/I	3	1.33 ± 0.58	2.3 ± 2.5	1.76 ± 0.28	110.86 ± 31.85
*Nepenthes tentaculata*	Borneo & Sulawesi	L	3	1.67 ± 0.58	4.2 ± 0.4	1.04 ± 0.62	41.01 ± 7.01
*Nepenthes truncata*	Philippines	U	4	1.25 ± 0.50	3 ± 1.4	1.78 ± 0.12	137.73 ± 21.31
*N*. × “Bill Bailey” (*N. singalana* × *ventricosa*)	Unnatural Hybrid, *N. ventricosa = *Philippines	U	6	2 ± 0.63	2.3 ± 0.9	1.86 ± 2.05	117.99 ± 20.22

For pitcher morph, L = “lower pitchers”,

U = “upper pitchers”, and I = “intermediate pitchers”, indicating the pitcher morph(s) representing that particular species in our dataset. ^a^One individual plant produced upper pitchers and the other produced lower pitchers in this case. ^b^In this case, all successfully extracted fluid samples were from lower pitchers, however a single upper pitcher of *N. maxima* was also collected.

### Sampling design

**Figure 1:**
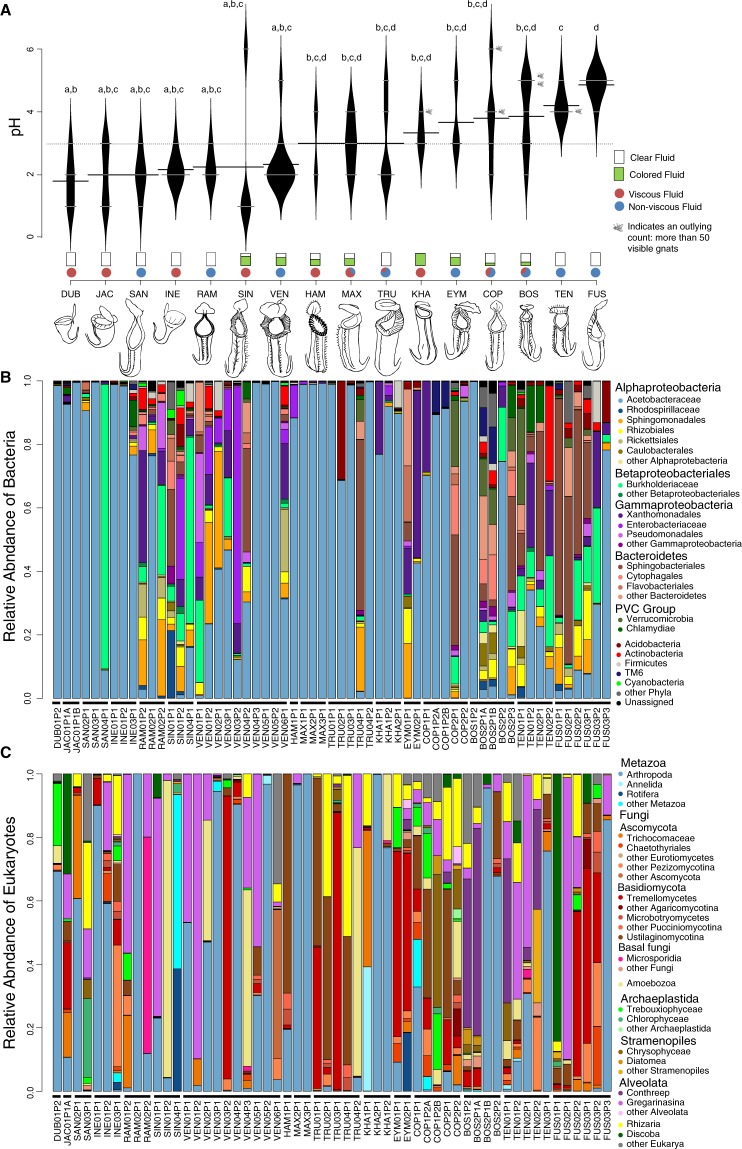
Illustration of fluid properties and community composition of experimental pitchers. (**A**) Beanplot representing pH levels of each species. The width of short white lines represents the number of samples at each value; long black lines represent means. Letters above indicate significant differences: species sharing a letter do not have significantly different means from one another. Small barplots represent portion of pitchers per species that exhibited colored fluid. Circles represent portion of pitchers per species that produced viscous fluid. Fly icons represent individual pitcher samples that contained more than 50 visible gnats. Illustrations of pitchers from each species by LSB; illustrations are not to scale and are for representative purposes only. Species are organized by mean pH. Species codes: DUB = *Nepenthes dubia*, JAC = *N. jacquelineae*, SAN = *N. sanguinea*, INE = *N. inermis*, RAM = *N. ramispina*, SIN = *N. singalana*, VEN = *N*. × “Bill Bailey”, HAM = *N. hamata*, MAX = *N. maxima*, TRU = *N. truncata*, KHA = *N. khasiana*, EYM = *N. eymae*, COP = *N. copelandii*, BOS = *N. boschiana*, TEN = *N. tentaculata*, FUS = *N. fusca* (**B**) Stacked barplot representing relative abundances of bacterial taxa. Note that “Rhodospirillaceae” here is Rhodosprillaceae *sensu lato* and also includes Reyranellaceae. (**C**) Stacked barplot representing relative abundances of eukaryotic taxa.

Sampling took place in July 2016. We aimed to sample from species that were represented by 2-3 individual plants and where possible, we sampled from 2 or 3 pitchers per individual (see Table 1 for full list of sampled plant species with successful extractions). We chose healthy pitchers of comparable size and age (noted by nodal position on the plant); however, the exact ages and time of opening were not known. For individual plants with multiple pitcher samples, we noted the ages of the pitchers relative to each other. In natural conditions, there may be successional changes in community composition with pitcher age^58–60^, however we tested the effect of relative pitcher age in our study and found that samples did not cluster by relative pitcher age (Mantel tests, bacteria: r = −0.05, p = 0.86; eukaryotes: r = 0.02, p = 0.31). The highly controlled nature of this experimental setup likely reduced the impact of successional effects, especially considering the similar ages of all plants used and the constancy of the environment to which they were exposed. We collected the entire fluid contents into sterile Falcon tubes using sterile Pasteur pipettes, wearing gloves as a further safeguard against contamination. Fluid pH was measured by placing a small drop of remnant fluid from the pipette directly onto a Millipore ColorpHast (0–14) indicator strip. Indicator strips provide a resolution of 1 pH unit, which is somewhat coarse (e.g. a sample with a reading of pH 3 on a strip may actually fall somewhere between 3 and 4 if using a finer resolution); nevertheless, this scale was suitable for our study as the range of pH values was wide (recall that pH is on a logarithmic scale). To preserve the DNA, we added 1 mL of Cetyl trimethylammonium bromide (CTAB) solution for every 1 mL of pitcher fluid^10^. The volume and color (e.g. clear or brightly yellow/green/pink-colored) of the fluid sample was also noted prior to addition of CTAB (Table 1, Fig. 1A). Fluid samples that had the property of forming unbroken strands between the pitcher and pipette tip during collection were considered to be viscoelastic (hereafter “viscous” for brevity)^39^. After DNA was extracted from the sample, solid particles (prey contents) were filtered out of the fluid using sterile gauze and the contents were examined under a dissecting microscope, identified, and counted. Prey counts were determined based on a combination of head capsules and wings.

### Sequencing and data processing

We used a metabarcoding approach to sequence the 16S and 18S rRNA genes in the fluid to represent the entire prokaryotic and eukaryotic communities in the pitcher fluid. DNA extractions were done as described previously^55^. First, to concentrate cells from preserved pitcher fluid we did an isopropanol precipitation, adding an equal volume of isopropanol to 1 mL (or less if the sample had lower volume) of preserved fluid and centrifuging at max speed for 10 minutes. The supernatant was discarded, and a buffer and phenol-chloroform were added before bead-beating at maximum speed for 2 minutes. We then extracted DNA from the liquid using a phenol-chloroflorm extraction method^61^. Negative controls were included for each set of extractions, and no measurable DNA was recovered from them. Amplicons were generated and sequenced at the Environmental Sample Preparation and Sequencing Facility (ESPSF) at Argonne National Laboratory. We targeted the V4 region of the 16S rRNA gene using primers 515F-806R^62,63^ and the V9 region of the 18S genes using primers Euk1391f-EukBr^64,65^. Primers were adapted with constructs designed for the Illumina platform. For the PCRs, each 25 µl reaction contained 9.5 µL of MO BIO PCR Water (Certified DNA-Free), 12.5 µL of QuantaBio’s AccuStart II PCR ToughMix (2x concentration, 1x final), 1 µL Golay barcode tagged Forward Primer (5 µM concentration, 200 pM final), 1 µL Reverse Primer (5 µM concentration, 200 pM final), and 1 µL of template DNA. The PCR conditions were: 94 °C for 3 minutes to denature the DNA, 35 cycles at 94 °C for 45 s, 50 °C for 60 s, and 72 °C for 90 s; and a final extension of 10 min at 72 °C. Amplicons were quantified using PicoGreen (Invitrogen) and the 16S and 18S sets were each separately pooled into single tubes in equimolar amounts. These pools were cleaned with AMPure XP Beads (Beckman Coulter) and quantified with a fluorometer (Qubit, Invitrogen). After quantification, the pools were diluted to 2 nM, denatured, and then diluted to final concentrations of 6.75 pM with a 10% PhiX spike. The 16S and 18S pools were sequenced on two separate Illumina MiSeq runs.

Sequences were assembled and assigned to operational taxonomic units (OTUs) using QIIME version 1.9 on Harvard’s Odyssey computer cluster^55^. Briefly, we joined forward and reverse reads using fastq-join, then split libraries with a PHRED quality cut-off of 20 to remove low-quality sequences, and used UCLUST (version 1.2.21q) open-reference clustering to form groups of sequences into OTUs with 97% similarity. We used the SILVA database version 132 and the UCLUST method for taxonomic classification of 16S and 18S OTUs. In some cases, further taxonomic assignment was determined using NCBI BLAST. Neighbor-joining phylogenies were constructed for all bacterial (16S) and eukaryotic (18S) OTUs. Samples were rarefied to 1328 (16S) and 1675 (18S) sequences for downstream community analyses. 16S OTUs classified as chloroplast and mitochondrial sequences, and 18S OTUs classified as Embryophyta (land plant) sequences were removed from downstream analyses of community composition to avoid inclusion of possible contaminants from host plant cells. Additionally, we repeated certain analyses for eukaryotes after removing all OTUs classified as fungus gnats (Insecta: Diptera: Sciaridae), in order to probe whether trends in community composition were sensitive to the high levels of prey DNA in some samples.

### Statistical analyses

All analyses were conducted in R version 3.5.0. In addition to testing for correlations between traits, we tested for phylogenetic signal in traits using previously published phylogenetic data^21^, the Pagel lambda^66^ and Blomberg *et al*. K^67^ statistic for continuous traits, and the Fritz and Purvis D^68^ statistic for qualitative traits. We used the function ‘betadisper’, paired with ‘permutest’ to calculate the homogeneity of variance in community composition across species. Some samples were excluded in order to achieve homogeneity of variance for analysis of community composition. We excluded *N. dubia* and *N. hamata* from our analysis of community composition, each of which had only a single sample from one pitcher that was successfully extracted. For eukaryotes, *N. jacquelineae* also had only a single successful extraction and was also excluded from ordinations and downstream analyses. Lastly, we excluded the hybrid taxon *N*. × “Bill Bailey” (*N. singalana* × *ventricosa*) for both bacterial and eukaryotic community composition analyses, due to its violation of homogeneity of variance and in order to limit the comparison of community structure to well recognized species rather than commercially produced hybrids.

Since it was not possible to extract DNA successfully from all of the samples and some samples returned sequences only for either 16S or 18S, we also performed logistic regressions (generalized linear models, binomial family with “logit” link) to assess potential biases in PCR amplification success as a consequence of pitcher properties. We used the ‘glmer’ function in the ‘lme4’ package^69^ and included all examined factors (including prey count, fluid color, fluid viscosity, and pH) as fixed effects in a single model with PCR amplification success as the predictor (16S and 18S each examined separately), with individual plant as a random effect.

Using the ‘vegan’ package, we assessed community-level similarity using the non-metric multidimensional scaling (NMDS) ordination method and the unweighted Unifrac distance metric. We assessed the significance of clustering by pitcher traits (species, pH, fluid color, and viscoelasticity) using the ‘adonis’ function in the ‘vegan’ package^70^, which conducts a specialized PERMANOVA test. For quantitative traits such as pH, we also performed a Mantel test. We further conducted canonical correspondence analysis (CCA), a form of constrained ordination, in order to more directly test the correlation between community composition and pH. We calculated alpha diversity according to the Shannon Index using the function ‘diversity’ in the ‘vegan’ package.

In order to examine patterns of differential abundance of individual OTUs in relation to fluid properties, we performed ANCOM (Analysis of Composition of Microbiomes)^71^, a test designed to examine taxon abundance while accounting for the fact that metagenomics studies yield relative abundance data as opposed to absolute abundance; one advantage of this test is that it can reveal changes in differential abundance of rare OTUs that otherwise do not affect community-level properties. For ANCOM tests, we used the full set of successfully extracted samples (including singleton species and the hybrid *N*. × “Bill Bailey”), only included OTUs with sequence counts above 100, and corrected for multiple testing (FDR) at a significance level of 0.05.

### Ethics approval

This article does not contain any studies with human participants or animals performed by any of the authors.

## Results

### Fluid properties and community composition

The bacterial communities found in our samples consist of several phyla, predominantly Proteobacteria (Alpha-, Beta-, and Gammaproteobacteria) and Bacteroidetes. Within Alphaproteobacteria, the family Acetobacteraceae was particularly dominant in some samples (comprising as much as 99.2% of sequences in a given sample, Fig. 1B). The eukaryotic communities found in our samples consist of a diverse assemblage of Metazoa, Fungi, Amoebozoa, Archaeplastida, Stramenopiles, Alveolata, Rhizaria, and Discoba (Fig. 1C).

The mean pH was significantly different across species (Kruskal-Wallis, χ^2^ = 42.98, p«0.001, Fig. 1A). Some species (*N. dubia*, *N. jacquelineae*, *N. sanguinea*, *N. inermis*, and *N. ramispina*) had a low mean pH (~2) and a narrow range (Table 1); *N. singalana* and *N*. × “Bill Bailey” also had a low mean pH, but with at least one high pH outlier each. Most of the remaining species had a more moderate mean pH (~3–4) and a very wide range of values, while *N. fusca* had a relatively high mean pH (4.86) and narrow range (Table 1). *N. fusca*’s pH is significantly different from that of *N. dubia*, *N. inermis*, *N. jacquelineae*, *N. ramispina*, *N. sanguinea*, *N. singalana*, *N. tentaculata*, and *N*. × “Bill Bailey” (posthoc Dunn Test with Benjamini-Hochberg correction, p < 0.05 for all pairs, Fig. 1A). We found no phylogenetic signal in mean pH, minimum pH, maximum pH, or pH range (Pagel’s lambda and Blomberg’s K, p > 0.10 in all cases; Supplementary Figure) for the species tested.

The different species also varied in their ability to produce viscous fluids: *N. sanguinea*, *N. ramispina*, *N*. × “Bill Bailey”, *N. eymae*, *N. tentaculata*, and *N. fusca* did not display the ability to do so (Fig. 1A). A few species produce colored fluid in some pitchers, including *N. singalana*, *N*. × “Bill Bailey”, *N. hamata*, *N. maxima, N. khasiana*, *N. eymae*, *N. copelandii*, and *N. boschiana* (Fig. 1A). We observed colored fluid in some unopened or newly opened pitchers from different (unsampled) individuals in the HortPark glasshouse, including members of our study species (data not shown), thus suggesting that the fluid coloration is largely plant-produced rather than a function of external inputs from the environment. Colored fluids were either reddish/pinkish/orange or yellowish/greenish (interestingly, we observed that initially yellowish/greenish samples instantly turned reddish/pinkish/orange upon the addition of CTAB, hinting at a common chemical nature of all colored fluid samples in our study).

The species did not differ significantly in prey capture (visible fungus gnat abundance, Kruskal-Wallis test, p > 0.05). Regarding correlations between traits, viscous pitcher samples tended to be more acidic (Kruskal-Wallis test, χ^2^ = 6.20, p = 0.012), and pitchers with visible prey tended to have a lower pH (Kruskal-Wallis test, χ^2^ = 5.03, p = 0.025). Colored fluid samples did not differ from clear fluid samples in pH or viscosity or prey capture (Kruskal-Wallis test, p > 0.05 in all cases); however, clear fluid had a disproportionately higher number of samples without visible prey (logistic regression, p = 0.003). We found no phylogenetic signal in presence of colored fluid (Fritz and Purvis’s D = −0.14, p = 0.08) or viscous fluid (Fritz and Purvis’s D = 0.74, p = 0.32; Supplementary Figure).

To assess potential biases in PCR amplification success, we tested for correlations between PCR amplification success and different fluid properties; we found some moderately significant correlations (Table 2). A greater number of visible gnats increased the likelihood of PCR amplification success for 16S (logistic regression, p = 0.03); also, 16S PCR amplification success increased with increasing pH (logistic regression, p = 0.03). Viscous samples were more likely to fail for 18S (logistic regression, p = 0.04).

**Table 2:** Results of logistic regressions performed on PCR amplification success (binary categorization of whether or not a sample successfully yielded detectable 16S/18S sequences) against prey counts (number of visible fungus gnats in pitcher), fluid color (clear or colored), viscosity (viscous vs. non-viscous), and pH level, within a generalized linear mixed model with plant as a random effect. N = 85 fluid samples.

Factor	16S			18S		
	Estimate	Standard Error	p-value	Estimate	Standard Error	p-value
Prey counts	0.341	0.1540	0.0269*	0.005	0.0122	0.6929
Fluid color	0.586	0.6882	0.3944	1.276	0.7373	0.0835
Viscosity	−0.699	0.5820	0.2299	−1.219	0.5923	0.0395*
pH	0.527	0.2468	0.0328*	0.494	0.2588	0.0562

*Indicates p < 0.05.

### Influence of pitcher fluid traits on community composition and alpha diversity

#### Nepenthes species

**Figure 2:**
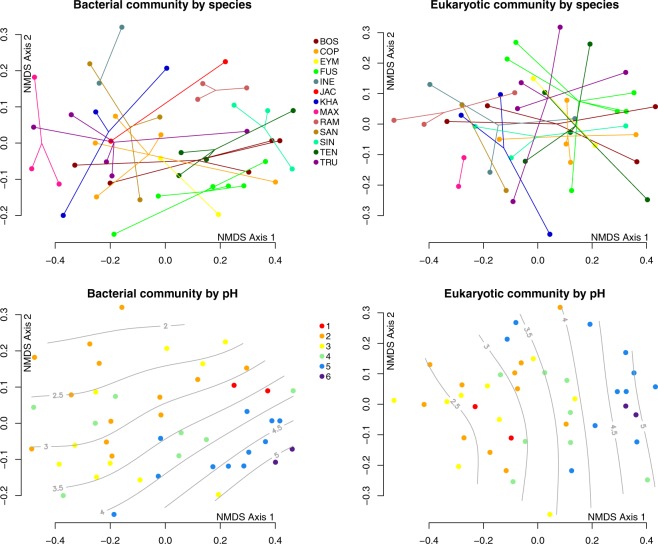
Non-metric multidimentional scaling (NMDS) plots representing community similarity (UniFrac distances) of bacteria (**A,C**) and eukaryotes (**B,D**) by host plant species identity (**A,B**) and by pH (**C,D**). Each point represents a sample and distance between points represents degree of similarity in community composition. Lines in A and B connect points belonging to the same species. Grey contour lines in C and D are smooth surfaces calculated based on variation in pH using the ‘ordisurf’ function in the ‘vegan’ package of R. Species codes: BOS = *N. boschiana*, COP = *N. copelandii*, EYM = *N. eymae*, FUS = *N. fusca*, INE = *N. inermis*, JAC = *N. jacquelineae*, KHA = *N. khasiana*, MAX = *N. maxima*, RAM = *N. ramispina*, SAN = *N. sanguinea*, SIN = *N. singalana*, TEN = *N. tentaculata*, TRU = *N. truncata*. pH levels: 1 = red, 2 = orange, 3 = yellow, 4 = green, 5 = blue, 6 = purple.

Both bacteria (PERMANOVA, R^2^ = 0.39, p < 0.001) and eukaryotes (PERMANOVA, R^2^ = 0.32, p = 0.004) show significant clustering by host species; however, a pairwise Adonis test shows no significant differences between individual species pairs (with Benjamini-Hochberg correction, p > 0.05 for all pairs; Fig. 2). In analyses of alpha diversity, bacterial communities differ in the mean and range of sample Shannon index by species; these means appear to be significantly different (Kruskal-Wallis test, χ^2^ = 29.637, p < 0.001), but individual species pairs are not significantly different under a Benjamini-Hochberg corrected post hoc Dunn test (p > 0.05 for all pairs). For eukaryotes, no significant differences exist in alpha diversity across species (Kruskal-Wallis test, χ^2^ = 18.38, p = 0.07). We found no differences in the resulting trends when we repeated these analyses with fungus gnat OTUs removed.

#### Fluid pH

We found a significant relationship between community composition and pitcher fluid pH for both bacteria (Mantel test, r = 0.23, p < 0.001) and eukaryotes (Mantel test, r = 0.32, p < 0.001). The CCA test also revealed that community composition changed as a function of changing pH levels, highly significant for both bacteria (χ^2^ = 0.62, p < 0.001) and eukaryotes (χ^2^ = 0.62, p < 0.001). Alpha diversity increases with increasing pH for both bacteria (linear regression, R^2^ = 0.27, p ≪ 0.001) and eukaryotes (linear regression, R^2^ = 0.39, p ≪ 0.001). When we repeated these analyses for eukaryotes with fungus gnat OTUs removed, we found no differences in the resulting trends.

#### Fluid color

Fluid color showed significant clustering for eukaryotes (PERMANOVA, R^2^ = 0.04, p = 0.03) but not bacteria (PERMANOVA, R^2^ = 0.025, p = 0.23). We repeated the analysis for eukaryotes with all OTUs assigned as fungus gnats removed, and in that case found no significant effect for eukaryotes (Adonis R^2^ = 0.0374, p = 0.07). Alpha diversity was not significantly different between colored and clear fluid samples for bacteria (Kruskal-Wallis test, χ^2^ = 0.56, p = 0.46). For eukaryotes, alpha diversity tended to be lower in colored fluid samples (Kruskal-Wallis test, χ^2^ = 6.13, p = 0.013); however, fluid color had no significant effect on alpha diversity if fungus gnats are removed (Kruskal-Wallis test, χ^2^ = 3.10, p = 0.078).

#### Viscosity

Fluid viscosity showed no significant clustering for either bacteria (PERMANOVA, R^2^ = 0.024, p = 0.26) or eukaryotes (PERMANOVA, R^2^ = 0.028, p = 0.22). Viscous samples had lower alpha diversity for both bacteria (Kruskal-Wallis test, χ^2^ = 4.43, p = 0.035) and eukaryotes (Kruskal-Wallis test, χ^2^ = 4.26, p = 0.039), though the effect was relatively weak. If fungus gnats are removed from the eukaryotic OTU table, viscosity had no effect on eukaryotic alpha diversity (Kruskal-Wallis test, χ^2^ = 2.88, p = 0.089).

### Influence of pitcher fluid traits on individual OTUs

#### Fluid pH

**Figure 3:**
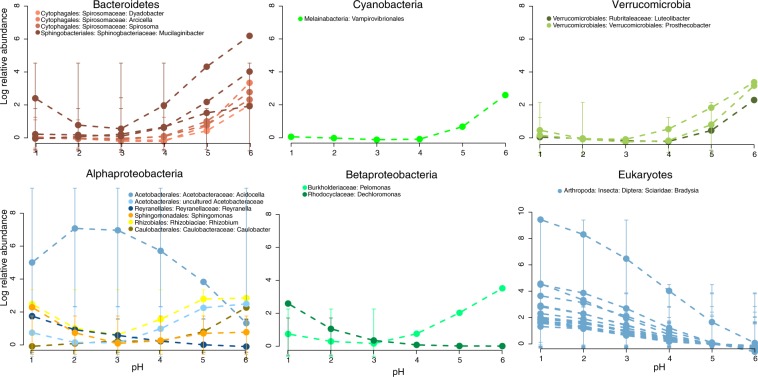
Results of analysis of composition of microbiomes (ANCOM) test, showing OTUs that are differentially abundant across pH levels. The points and dotted lines are smooth splines generated to summarize the individual trends in change of mean log relative abundance for each OTU across pH levels. Bars indicate standard deviation.

According to the ANCOM test, the abundances of 25 bacterial and 17 eukaryotic OTUs were significantly differentially abundant across the different pH levels of the pitcher fluids. Bacteria exhibited variation in individual OTU response to pH level, with most OTUs increasing in abundance with increasing pH (all OTUs within Bacteroidetes, Cyanobacteria, and Verrucomicrobia), and others decreasing with increasing pH (Fig. 3). The minority of OTUs that decreased with increasing pH include *Acidocella* (Acetobacteriaceae) and *Reyranella* (Reyranellaceae, formerly Rhodospirillaceae^72,73^). An uncultured Acetobacteriaceae OTU exhibits the opposite pattern from its confamilial *Acidocella*. Most other OTUs in Alphaproteobacteria generally increased with increasing pH, except *Sphingomonas*, which generally decreased with increasing pH. In the class Betaproteobacteria (which a recent study proposes may be recircumscribed as an order within Gammaproteobacteria^72^), one OTU (*Dechloromonas*) decreased with increasing pH, while the other one (*Pelomonas*) increased with increasing pH. All significant eukaryotic OTUs decreased with increasing pH, and all are assigned as fungus gnats (Diptera: Sciaridae: Bradysia).

#### Fluid color

**Figure 4:**
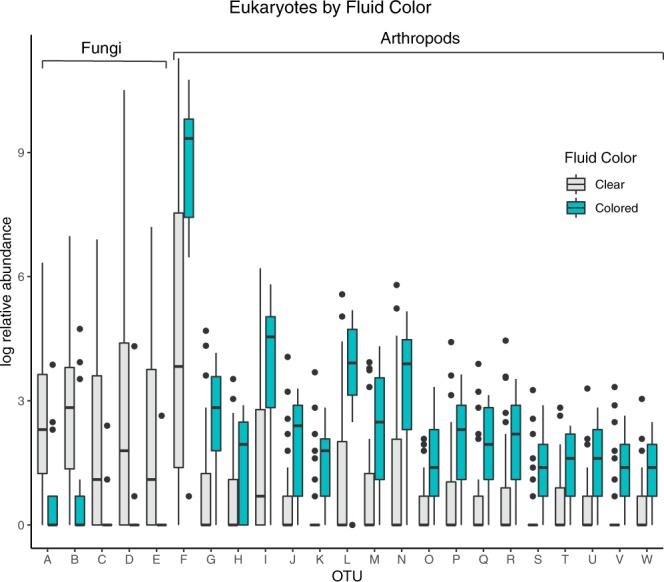
Results of analysis of composition of microbiomes (ANCOM) test, showing OTUs that are differentially abundant between the two categories of fluid color (clear and colored). The individual OTU taxonomic assignments are as follows: A-B: “Ascomycota: Pezizomycotina: Eurotiomycetes: Chaetothyriales: *Capronia*”, C: “Basidiomycota: Agaricomycotina: Tremellomycetes: *Tremella*”, D: “Basidiomycota: Agaricomycotina: Tremellomycetes: *Syzygospora*”, E: “Basidiomycota: Pucciniomycotina: Microbotryomycetes: *Rhodosporidium*”, F–W: “Arthropoda: Insecta: Diptera: Sciaridae: *Bradysia*”.

The ANCOM test reveals 23 eukaryotic OTUs differentially abundant by fluid color: 5 OTUs in Fungi and 18 OTUs in Metazoa (Fig. 4). The metazoan OTUs were more abundant in colored fluid (most of which are absent from clear fluid); conversely, the fungal OTUs are more abundant in clear fluid (completely absent from colored fluid). The abundance of only a single bacterial OTU was significantly correlated with fluid color, an unassigned Enterobacteriaceae, which was more abundant in colored than clear fluid.

#### Viscosity

The ANCOM test shows that the abundance of only a single bacterial OTU, an uncultured Lachnospiraceae (Firmicutes: Clostridiales), was significantly correlated with fluid viscosity, and it was more abundant in viscous fluid than non-viscous fluid. Only a single eukaryotic OTU had a significant ANCOM result by viscosity, *Chlamydomonas* (Archaeplastida: Chlorophyceae), and it was more abundant in viscous fluid than non-viscous fluid.

## Discussion

We investigated how *Nepenthes* pitchers might act as ecological filters, especially via their manipulation of the abiotic properties of their fluid. In order for *Nepenthes* species to affect the ability of microbes to establish and persist in their pitchers, they must be able to alter their fluid properties. We demonstrated that the various species in our experiment do in fact alter their fluid properties, including pH modification and the production of viscous and/or colored fluid. The pitchers in our study were all filled with the same pH 6.5 water at the beginning of the experiment and yet after about two weeks of acclimation, they ended with a pH range spanning from ~1–6, and clear differences in fluid viscosity and color. We designate the differences in fluid properties as caused by both biotic (fluid viscosity, color) and abiotic factors (pH); however, the designation is not clear cut. The pH level, like viscosity or fluid color, is largely a function of pitcher physiology, and accordingly shows interspecific variation (Fig. 1A). This level of variation is striking: even with relatively low replication within species, it is clear that species differ in their characteristic pH range, with some species being more stable than others. For example, out of 12 pitchers of *N*. x “Bill Bailey”, 10 were pH 2 and out of 7 pitchers of *N. fusca*, 6 were pH 5 (Fig. 1A).

We first tested how *Nepenthes* species identity shapes variation in the community compositions of organisms housed within pitchers. At a first approximation, the magnitude of the effect of host species identity on community composition is similar to that of pH; however, a *post hoc* test reveals that with the number of species involved, the differences between any species pair taken in isolation lacks statistical significance. To further probe the role of species identity as a force in shaping microbial assembly, we performed the ANCOM test for the presence of significantly differentially abundant OTUs within *N. ramispina* (a species with a visibly separated cluster in the ordination for bacteria, Fig. 2) versus all other species. While *N. ramispina* did contain OTUs that were unique or differentially abundant with respect to all other species pooled, these were OTUs that did not differ across the other species that share a similar pH mean and range (low and narrow, Fig. 1A). Hence these OTUs were likely associated with *N. ramispina*’s particular pH regulatory properties rather than *N. ramispina* itself. The fact that physiologically similar *Nepenthes* species do not contain characteristic and significantly different OTUs further suggests that trait variation rather than species identity *per se* is the factor that acts as an ecological filter in this study. This supports pH as the primary factor of importance among the traits we measured for community assembly within *Nepenthes* pitchers.

The influence of pH on both bacteria and eukaryotes is strong, both in terms of community composition and in terms of the dynamics of individual OTUs (as seen in ANCOM results, Fig. 3). Most bacterial OTUs are less abundant in highly acidic fluid, and the overall alpha diversity is lower as well. This speaks to the harshness of low pH conditions, where only a few specialized acidophiles are able to thrive, such as the Acetobacteraceae. Interestingly, species in the Acetobacteraceae, especially of the genus *Acidocella*, appear to be common associates of *Nepenthes*, not only in this study, but in wild samples as well (Supplemental Discussion).

For eukaryotes, all OTUs with significant differential abundance at different pH levels were found to decrease with increasing pH. All of these OTUs were assigned as fungus gnats (Insecta: Diptera: Sciaridae) by BLAST. The high numbers of fungus gnat sequences at low pH levels may have multiple non-exclusive explanations: (1) prey capture has been recorded in other species to lower pH by inducing fluid acidification^74,75^; (2) pitchers belonging to high-acidity species may also be more successful at prey capture, assuming no bias in fungus gnat occurrence throughout the glasshouse; and/or (3) acidic pitchers may also be better at digestion, thus leaving less physical evidence of their prey capture success. In addition to these sources of intra-specific variability, pH differences are largely explained by species differences, as even with prey induction, it has been demonstrated that not all species are capable of achieving the same levels of acidity^75^. Also, the samples with the greatest number of visible gnats had relatively moderate to high pH (pH 4–6, Fig. 1). Future studies examining how prey abundances in pitchers correlate to 18S rRNA sequence counts could help to clarify this^10^. The effect of pH on eukaryotes is likely not limited to effects on prey DNA, though, as pH still has a significant effect on eukaryotic community composition after removing fungus gnats from the OTU table. Microbial eukaryotes living symbiotically in the fluid such as fungi, algae, and amoebae likely experience physiological challenges in acidic conditions similar to the bacteria, and/or appear to be similarly affected due to their interactions with the bacteria themselves. Future experimental work can distinguish direct effects of pH on microbial taxa, effects due to interactions between taxa, and effects due to prey.

It was surprising that viscosity, a biotic factor and definitively plant-regulated trait, had only a weak relationship to community structure, with no significant difference in community composition for either bacteria or eukaryotes between species with different fluid viscosities. The only effect we noted was that viscous pitchers had lower alpha diversity for both bacteria and eukaryotes. This might suggest that viscous fluid presents a harsher environment for inquilines, similar to how low pH environments lead to reduced diversity. Notably, 18S extractions from viscous samples were more likely to fail than those from non-viscous samples (Table 2). This was similar to lower 16S PCR amplification success for more acidic fluids (Table 2), mirroring the trend of decreasing bacterial alpha diversity with increasing acidity. However, without qPCR data directly measuring numbers of ribosomal RNA genes, it is not possible to ascertain whether PCR amplification failure can be attributed to reduced microbial abundance or to some form of bias introduced by the extraction process. In any case, viscosity might have a larger impact on individual OTUs than on community composition, as our ANCOM results revealed OTUs with significant differential abundances between viscous and non-viscous fluids. However, it should be noted that we determined viscosity visually and recorded it as a binary trait^39^; quantitative rheological measurements^40^ might have revealed continuous variation in viscoelasticity across samples, which may have had more explanatory power. As another caveat, levels of viscosity in this experiment possibly differ from natural conditions, as viscosity can be plastic^76,77^.

For eukaryotes, but not for bacteria, colorful pitcher fluids had significantly lower alpha diversity than clear fluids, suggesting fluid color to be a more important factor for eukaryotic communities. However, the effects of fluid color on eukaryotic community composition and alpha diversity are not robust, and disappear when fungus gnats are removed from the dataset. Like viscosity, fluid color appears to be more important at the individual OTU level than it is at the community level. Fluid color could be an indication of the production of droserone and 5-O methyl droserone. Past studies have shown that the presence of these compounds results in reddish^37,78^ or yellowish^78^ fluid coloration. Droserone and 5-O methyl droserone are anti-fungal agents induced by prey capture, specifically in response to chitin^37,78,79^. This could explain the higher abundance of fungus gnat DNA in colored samples relative to clear fluid samples, accompanied by a decrease in the relative abundance of certain fungal OTUs (Fig. 4). Without confirmation by chemical analyses like Gas Chromatography-Mass Spectrometry (GCMS) this explanation remains somewhat speculative, but the pattern is suggestive. Pitcher fluid coloration is not a well-documented trait in the literature; to our knowledge, plant-produced colored fluid has only been reported for *N. khasiana*^37,78,79^. Our observation of colored fluid in several species is novel, and future work should investigate this this trait, both in the field and in cultivation, as it might impact fungal colonization and survival.

Interspecific differences in pH regulation may be linked to functional/ecological differences between species. Future work should determine what ecological functions link the species that regulate their fluid pH levels in similar ways. In addition to interspecific trait variation, intraspecific variation is characteristic of *Nepenthes*. Most species produce two distinct pitcher morphs, “lower pitchers” from the terrestrial rosette phase and “upper pitchers” from the aerial climbing phase, occasionally with intermediate morphs produced during the transition between growth phases^22^. In some species the morphological and ecological differences between lower and upper morphs can be quite extreme, e.g. the insectivorous lower and coprophagous upper pitchers of *N. lowii*^23^. Thus, it may be valuable to examine between-morph fluid trait differences within species in future studies. Unfortunately, we were unable to adequately sample both morphs within species in order to meaningfully compare fluid traits between morphs; our species are generally represented by single morphs (Table 1).

In this study, it was not possible to assess the functional significance of differentially abundant microbial OTUs, but these could be probed by future transcriptomic or proteomic work. The observation of certain OTUs frequently occurring in *Nepenthes* pitchers in both natural and artificial situations could indicate that these particular associations are ecologically significant, so the common *Nepenthes* symbionts like *Acidocella* found here merit further research from a functional perspective. Pitchers may also modify other abiotic features of the fluid such as dissolved oxygen levels or temperature, so additional fluid properties should be examined in future work as well.

Our research supports the hypothesis that *Nepenthes* pitcher plants regulate abiotic factors, potentially as a means of maintaining species-specific microbial associations. This is important in considering the possibility of community codiversification^80,81^. From the perspective of the host, as long as an abiotic factor is under host control, it functionally becomes an extended phenotype with the same potential for evolution in response to interspecies interactions^82^ as any other biotic phenotype. However, from the perspective of the microbial symbionts, evolution in response to host conditions becomes much less tight. Microbes that respond to a purely biotic factor, such as secondary metabolites, can be considered to be necessarily linked to the evolution of the host, as the exact biochemical compounds involved are unlikely to be found in other environmental contexts. On the other hand, when microbes respond to an abiotic factor, such as fluid pH, those microbes may have been pre-adapted to live in a wide range of environments that incidentally fit that factor, such as other small aquatic environments. So even if the abiotic factor is a product of host evolution in one context, the symbionts may not have evolved in response to the host. Thus, the evolutionary implications of biotic and abiotic filters can be quite different from the perspective of the symbiont, despite having similar implications from the perspective of the host.

## Supplementary information

Supplementary information.

## Data Availability

Data files and R code associated with this publication will be made available on Harvard Dataverse (10.7910/DVN/GXXKU0). Raw sequences (16S and 18S) will be uploaded to the NCBI Sequence Read Archive (SRA) https://www.ncbi.nlm.nih.gov/bioproject/PRJNA605027.
