# Supplementary material for: Tropical pitcher plants (Nepenthes) act as ecological filters by altering properties of their fluid microenvironments
Source: Sci Rep. 2020 Mar 10;10:4431. doi: 10.1038/s41598-020-61193-x (PMC7064508; doi:10.1038/s41598-020-61193-x)
Supplement: Supplementary file 1 — Supplementary information. [file 41598_2020_61193_MOESM1_ESM.pdf]

Title: Tropical pitcher plants (*Nepenthes*) act as ecological filters by altering properties of their fluid microenvironments

Authors: Kadeem J. Gilbert[a]*, Leonora S. Bittleston [a,b], Wenfei Tong[c], Naomi E. Pierce[a]

[a] Department of Organismic and Evolutionary Biology, Harvard University, 26 Oxford St., Cambridge, MA 02138, USA

[b] Department of Civil and Environmental Engineering, Massachusetts Institute of Technology, 77 Massachusetts Avenue, Room 1-290 Cambridge, MA 02139

[c] Department of Biological Sciences, University of Alaska, Anchorage, 3211 Providence Drive, Anchorage, AK 99508

*Corresponding author: kgilbert@g.harvard.edu, (908) 251-2326

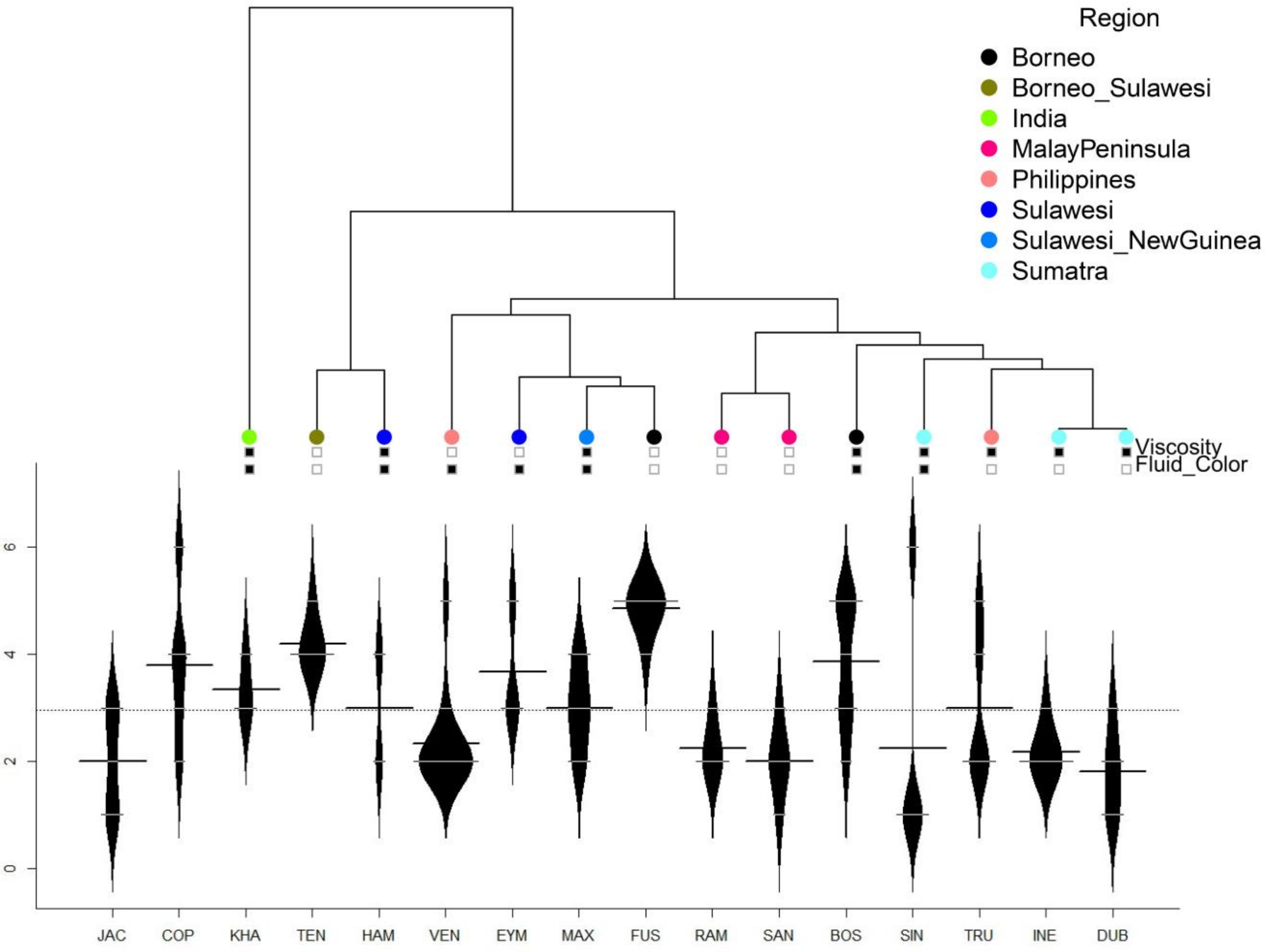

Supplementary Figure: Pitcher traits viewed in a phylogenetic context, using the Gilbert et al. (2018) topology for all previously sequenced species included in this study. *N. jacquelineae* and *N. copelandii* had not been sequenced; for the purpose of this illustration *N. x "Bill Bailey"* is coded according to parent species *N. ventricosa*. Beanplot shows pH by species; the species have been reorganized to fit the phylogeny. For viscosity and fluid color, a white square denotes absence and a filled in square denotes presence. Tips are additionally colored by the region of origin of each species. Species codes: JAC = *N. jacquelineae*, COP = *N. copelandii*, KHA = *N. khasiana*, TEN = *N. tentaculata*, HAM = *N. hamata*, VEN= *N. ventricosa*, EYM = *N. eymae*, MAX = *N. macima*, FUS = *N. fusca*, RAM = *N. ramispina*, SAN = *N. sanguinea*, BOS = *N. boschiana*, SIN = *N. singalana*, TRU = *N. truncata*, INE = *N. inermis*, DUB = *N. dubia*.

Supplemental Discussion: Taxonomic composition of experimental pitchers

Bacteria

Previous studies indicate a characteristic set of bacterial taxa associate with pitchers of *Nepenthes* species, as well as with the convergently evolved pitchers of New World pitcher plants in the genus *Sarracenia* (Bittleston et al. 2018). Some of the common bacterial taxa in our samples match what appear to be common associates of other pitcher plant taxa, especially the orders Acetobacterales (formerly under Rhodospirillales), Actinomycetales, and Rhizobiales, but also Sphingobacteriales (including the family Chitinophagaceae), Burkholderiales, Enterobacteriales, and Xanthomonadales (Bittleston 2018). While not dominant in terms of relative abundance, Acidobacteria and Caulobacterales occur fairly frequently across multiple samples, and these taxa have also been found to associate with *Nepenthes* in nature (Sickel et al. 2016; Bittleston 2018). Some of the common taxa in our study may also be common inhabitants of plant surfaces in general, such as Sphingomonadales, Pseudomonadales, and Xanthomonadales that have all been found in earlier studies of phyllosphere bacteria in other plant species (Vorholt 2012; Vacher et al. 2016). Other abundant or frequent taxa in our study include bacteria that do not appear to be frequent associates of pitchers, and these could represent environmental bacteria that are a consequence of the experimental glasshouse setting: Chlamydiae, Rickettsiales, TM6, and Verrucomicrobia. Most samples also possess a small fraction of OTUs that cannot be assigned at the phylum level. Overall, while the bacterial communities in our experimental plants may not be typical of those found in wild *Nepenthes*, we nevertheless recovered many commonalities in the diversity and abundance of certain groups.

Acetobacteriaceae, particularly the genus *Acidocella* (and to a lesser extent, *Acidisoma*) show up as frequent associates of *Nepenthes* pitchers in most microbiome studies so far, from natural or semi-natural settings (Chou et al. 2014; Kanokratana et al. 2016; Sickel et al. 2016; Bittleston et al. 2018). Considering *Acidocella* sp.'s apparent relationship with *Nepenthes* and given that it is not known to be a common environmental bacterium, previously isolated from only a few extreme habitats (Kishimoto et al. 1995; Belova et al. 2009; Kimoto et al. 2010; Jones et al. 2013), it would be worthwhile to probe the function of taxa from this genus in relation to its host.

The possibility of vertical transmission cannot be completely ruled out regarding important *Nepenthes* symbionts. Vertical transmission has been seen in other phyllosphere systems (Vorholt 2012), and although it was initially established that pitchers are sterile prior to opening (Buch et al. 2012), more recent microbiome studies were able to find bacterial DNA in unopened pitchers (Chou et al. 2014; Takeuchi et al. 2015; Kanokratana et al. 2016), including *Acidocella* (Chou et al. 2014; Kanokratana et al. 2016). In each case, only a minority of unopened pitchers examined yielded detectable amounts of DNA, so the possible occurrence of bacteria in unopened pitchers is unresolved. In known cases of vertical transmission in plants, seed-associated bacteria can spread systemically throughout the developing plant (Vorholt 2012), so it is interesting to note that Sickel et al. (2016) found that bacterial composition did not differ significantly between pitcher fluid, pitcher external surfaces, and leaf lamina in their study. As evidence against vertical transmission of *Acidocella* sp., a previous greenhouse *Nepenthes* microbiome study did not find *Acidocella* as a prominent taxon (Takeuchi et al. 2015). However, the Takeuchi et al. (2015) study took place in temperate Germany, and it seems likely that key associates of pitchers such as *Acidocella* sp. are themselves geographically restricted, which could explain its presence both in the wild and in the Singapore greenhouse, without needing to invoke vertical transmission.

Eukaryotes

Symbiotic arthropod communities in pitcher plants, including several families of dipteran larvae and mites, have been well-characterized over decades of research (Beaver 1979). Being in an enclosed glasshouse, however, our experimental plants exhibited no evidence of being colonized by symbiotic

arthropods. Additionally, microfauna such as nematodes and rotifers that may be expected to be fairly common members of wild pitcher communities (Quisado 2013; Bittleston et al. 2016; Bittleston 2018) were not common in our samples. Nevertheless, insect prey was available in the glasshouse, primarily fungus gnats (Sciaridae), which are common indoor plant pests that can be found in potting media. This prey DNA was detectable in our samples, enabling us to examine the impact of prey capture on microbiome dynamics.

Few studies have documented the composition of eukaryotic microbial communities in wild *Nepenthes* (Bittleston et al. 2016; Bittleston 2018; Bittleston et al. 2018). Fungi such as Saccharomycetes, Agaricomycetes, and basal fungi (e.g. Mucoromycotina, Chytridiomycota) have been previously found in wild (Bittleston 2018; Bittleston et al. 2018), and these taxa also were present in our samples. However, while ascomycetous yeasts in Saccharomycetes appear to be the dominant fungi in wild communities (Bittleston 2018), our samples were dominated by Basidiomycota in Ustilaginomycotina and in Agaricomycotina (especially Tremellomycetes). Tremellomycetes can exist in single-celled yeast form, so these fungi could hypothetically occupy a similar ecological niche to the fungal yeasts in wild pitchers. The Ascomycota in our samples include Trichocomaceae (including the well-known genus *Penicillium*), which are common members of indoor microbial communities (Barberán et al. 2015); Chaetothyriales ("black yeasts" including the family Herpotrichiellae), which were previously found in wild pitchers (Bittleston 2018); as well as Sordariomycetes and Leotiomycetes, also members of Pezizomycotina. A single pitcher sample was dominated by Microsporidia, a basal fungus and obligate intracellular parasite of animals. So while certain fungal taxa have been found in wild pitchers, the overall taxonomic composition of fungi in our study appears to have had some major influence from the microbial pool of the greenhouse environment.

Pitcher plant microbiomes in this and previous studies also include Protista. Algae have been found to be common inhabitants of pitchers in the wild (Bittleston et al. 2016, Bittleston 2018), and we found a diverse community of algae throughout our samples, including members of Archaeplastida (Trebouxiophyceae and Chlorophyceae), Stramenopiles (Chrysophyceae and diatoms), and Discoba (Euglenozoa). Rhizaria and Amoebozoa are two abundant taxa in our samples that have also been found associated with pitchers in other studies (Bittleston et al. 2016, Bittleston 2018). Another common taxon across our samples was Alveolata, particularly Gregarinasina (gregarines). These are obligate arthropod parasites, especially of larval mosquitoes (Chen 1999; Tseng 2007) and they have been previously found in pitcher communities in association with the symbiotic larval dipterans (Baker et al. 2016; Bittleston et al. 2016). As there were no mosquitoes or dipteran larvae of any kind inhabiting our pitchers, the frequent occurrence of gregarines here is somewhat surprising. These gregarines may have been parasitizing the adult fungus gnats trapped by the pitchers; this is supported by ANCOM analysis showing that gregarine relative abundance is higher in pitchers with visible prey than those without visible prey (see figure) Perhaps more culture-independent surveys of eukaryotic diversity will reveal that gregarines are an even more important component of ecosystems than currently appreciated, perhaps infecting a wide assortment of arthropods in a variety of ecological contexts (Dabert and Dabert 2008; Criado-Fornelio et al. 2017).

Overall, the eukaryotic community composition appears to be somewhat uneven at the broad taxonomic level compared to the bacteria. Multiple phyla generally can be seen co-occurring within most samples for bacteria. However, for eukaryotes, most samples appear to be dominated by a single broad taxon, i.e. fungus-, metazoan-, or protist-dominated communities.

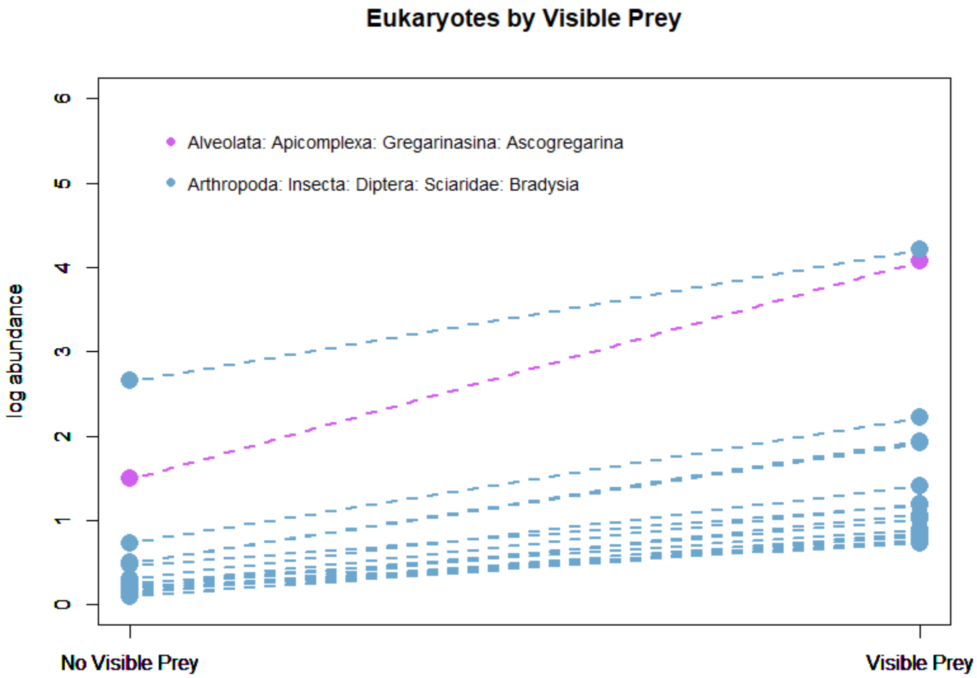

Figure: Results of ANCOM analysis showing slopes of change in log relative abundance for eukaryote OTUs in relation to presence of visible prey

References

Baker CC, Bittleston LS, Sanders JG, Pierce NE. **2016**. Dissecting host-associated communities with DNA barcodes. *Phil. Trans. R. Soc. B* **371**: 20150328.

Barberán A, Dunn RR, Reich BJ, Pacifici K, Laber EB, Menninger HL, Morton JM, Henley JB, Leff JW, Miller SL, others. **2015**. The ecology of microscopic life in household dust. *Proc. R. Soc. B* **282**: 20151139.

Beaver R. **1979**. Biological studies of the fauna of pitcher plants [*Nepenthes*] in west Malaysia. *Annales de la Société Entomologique de France*.3–17.

Belova SE, Pankratov TA, Detkova EN, Kaparullina EN, Dedysh SN. **2009**. *Acidisoma tundrae* gen. nov., sp. nov. and *Acidisoma sibiricum* sp. nov., two acidophilic, psychrotolerant members of the Alphaproteobacteria from acidic northern wetlands. *International Journal of Systematic and Evolutionary Microbiology* **59**: 2283–2290.

Bittleston LS. **2018**. Commensals of *Nepenthes* Pitchers, in *Carnivorous Plants: Physiology, Ecology, and Evolution*. Ellison, Aaron M and Adamec, Lubomír ed. Oxford University Press, 314–332.

Bittleston LS, Baker C, Strominger LB, Pringle A, Pierce NE. **2016**. Metabarcoding as a tool for investigating arthropod diversity in *Nepenthes* pitcher plants. *Austral Ecology* **41**: 120–132.

Bittleston LS, Wolock CJ, Yahya BE, Chan XY, Chan KG, Pierce NE, Pringle A. **2018**. Convergence between the microcosms of Southeast Asian and North American pitcher plants. *eLife* **7**.

Buch F, Rott M, Rottloff S, Paetz C, Hilke I, Raessler M, Mithöfer A. **2012**. Secreted pitfall-trap fluid of carnivorous *Nepenthes* plants is unsuitable for microbial growth. *Annals of Botany*: mcs287.

Chen W. **1999**. The life cycle of *Ascogregarina taiwanensis* (Apicomplexa: Lecudinidae). *Parasitology Today* **15**: 153–156.

Chou LY, Clarke CM, Dykes GA. **2014**. Bacterial communities associated with the pitcher fluids of three *Nepenthes* (Nepenthaceae) pitcher plant species growing in the wild. *Archives of Microbiology* **196**: 709–717.

Criado-Fornelio A, Verdú-Expósito C, Martin-Pérez T, Heredero-Bermejo I, Pérez-Serrano J, Guàrdia-Valle L, Panisello-Panisello M. **2017**. A survey for gregarines (Protozoa: Apicomplexa) in arthropods in Spain. *Parasitology Research* **116**: 99–110.

Dabert M, Dabert J. **2008**. Ribosomal DNA sequences reveal gregarine pathogens (Apicomplexa: Gregarinia) in mites and other arachnids (Arachnida). *Soil Org* **80**: 197–204.

Jones RM, Hedrich S, Johnson DB. **2013**. *Acidocella aromatica* sp. nov.: an acidophilic heterotrophic alphaproteobacterium with unusual phenotypic traits. *Extremophiles : Life Under Extreme Conditions* **17**: 841–50.

Kanokratana P, Mhuanthong W, Laothanachareon T, Tangphatsornruang S, Eurwilaichitr L, Kruetreepradit T, Mayes S, Champreda V. **2016**. Comparative Study of Bacterial Communities in *Nepenthes*. *Microbial Ecology* **72**: 381–393.

Kimoto K, Aizawa T, Urai M, Ve NB, Suzuki K, Nakajima M, Sunairi M. **2010**. *Acidocella aluminiidurans* sp. nov., an aluminium-tolerant bacterium isolated from *Panicum repens* grown in a highly acidic swamp in actual acid sulfate soil area of Vietnam. *International Journal of Systematic And Evolutionary Microbiology* **60**: 764–768.

Kishimoto N, Kosako Y, Wakao N, Tano T, Hiraishi A. **1995**. Transfer of *Acidiphilium facilis* and *Acidiphilium aminolytica* to the genus *Acidocella* gen. nov., and emendation of the genus *Acidiphilium*. *Systematic and Applied Microbiology* **18**: 85–91.

Quisado JS. **2013**. Nematode diversity of phytotelmata of *Nepenthes* spp. in Mount Hamiguitan Range Wildlife Sanctuary, Philippines. (thesis)

Sickel W, Grafe TU, Meuche I, Steffan-Dewenter I, Keller A. **2016**. Bacterial diversity and community structure in two Bornean *Nepenthes* species with differences in nitrogen acquisition strategies. *Microbial Ecology* **71**: 938.

Takeuchi Y, Chaffron S, Salcher MM, Shimizu-Inatsugi R, Kobayashi MJ, Diway B, von Mering C, Pernthaler J, Shimizu KK. **2015**. Bacterial diversity and composition in the fluid of pitcher plants of the genus *Nepenthes*. *Systematic and Applied Microbiology* **38**: 330–339.

Tseng M. **2007**. Ascogregarine parasites as possible biocontrol agents of mosquitoes. *Journal of the American Mosquito Control Association* **23**: 30–34.

Vacher C, Hampe A, Porté AJ, Sauer U, Compant S, Morris CE. **2016**. The phyllosphere: microbial jungle at the plant-climate interface. *Annual Review of Ecology, Evolution, and Systematics* **47**: 1–24.

Vorholt JA. **2012**. Microbial life in the phyllosphere. *Nature Reviews. Microbiology* **10**: 828–40.
